# A hybrid model for EEG-based gender recognition

**DOI:** 10.1007/s11571-019-09543-y

**Published:** 2019-07-04

**Authors:** Ping Wang, Jianfeng Hu

**Affiliations:** The Center of Collaboration and Innovation, Jiangxi University of Technology, Nanchang, 330098 China

**Keywords:** Gender recognition, Electroencephalogram (EEG), Entropy measures, Random forest (RF), Logistic regression (LR)

## Abstract

The gender recognition is an important research field to study evidence regarding some personal characteristics in the information and data society. However, some current traditional methods such as vision and sound have been exposed their own security weaknesses. Recently, biometric gender recognition based on Electroencephalography (EEG) signals has been widely used in information safety and medical fields. It is necessary to explore potential of using EEG to present a more robust and accurate result with larger training data based on sophisticated machine learning approaches. In this contribution, we present an automated gender recognition system by a hybrid model based on EEG data of resting state from twenty-eight subjects. These data are useful and handy to get insights into assessing the differences in personal gender. For achieving a good performance and a strong robustness, the system develops a hybrid model of combining random forest and logistic regression, and employs four common entropy measures to analyze the non-stationary EEG signals. Result also suggests that the recognition performance achieve an improved progress with an accuracy of 0.9982 and AUC of 0.9926 based on a nested tenfold cross-validation loop, implying that show a significant potential applicability of the proposed approach and is capable of recognizing personal gender.

## Introduction

Gender recognition using the cutting-edge computer approaches is a meaningful research topic for revealing personal characteristics in the information society (Udry 1994). The research of gender analysis is one of the most important fields with huge potential applications such as human–computer interaction (Beckwith et al. 2006), information security (Demirkus et al. 2010), commercial exploitation (Maldonado et al. 2003), cultural exploration (Gul and Humphreys 2014) and population research (Hoffmeyer-Zlotnik and Wolf 2003). Thus, promoting recognition technologies for distinguishing individuals according to their gender characteristics is critical and valuable.

Actually, there are two common ways for human beings to accurately and quickly recognize individual gender in daily life, sight and sound (Bruce et al. 1993). However, it is a difficult challenge for the automatic equipment or system to actively judge individual gender at present. With the rapid rise of information technology and artificial intelligence, it provided more opportunities to promote the gender recognition technologies based on computational methods (Miller 2013). Mendoza et al. (1996) discussed acoustical differences between male and female voices by using of the long-term average spectrum. The facial expression changes were also exploited to reveal gender differences in numerous studies recently. Azzopardi et al. (2016) proposed a novel descriptor based on COSFIRE filters for gender recognition from face images and achieved an accuracy rate of 93.7%. Ergen and Abut (2013) performed the gender classification based on front façade photos of 100 male and 100 female and obtained the highest achievement of 88%. In another work, Zhang et al. (2016) compared the encoded features from both 2D and 3D face images in order to achieve automatic gender recognition with an average high accuracy of 96.23%. Shan (2013) investigated gender recognition on real-life faces by using the Labeled Faces in the Wild and AdaBoost classifier to obtain the performance of 94.81%. In addition, some detailed reviews discussed and analyzed the gender recognition about face of humans by various methods (Ng et al. 2015; Rai and Khanna 2012). Besides, other parts of the body such as hand shapes, fingernails and fingerprints were also used to identify gender, but the stability of the results is not very good (Murdan 2011; Gnanasivam and Muttan 2012; Amayeh et al. 2008). Through the above, current approaches for gender recognition were largely determined by facial photographs.

However, there is very little investigation into gender recognition based on physiological measurements such as electrocardiogram (ECG) and electroencephalogram (EEG), which also can show different characteristics for individual gender (Moss 2010; Hu 2017). Due to the fact that there are some limitations for the existing methods about identifying gender, it is necessary to consider other feature methods especially physiological measurements. For example, the recognition for gender relied on body part features, most of which are face expression, involved in poor accuracy or personal privacy. Facial recognition also needs a large amount of sample datasets that computed with complex high-dimensional algorithms. In addition, there are application limitations in some specific areas such as senior crime and transsexual study (Bilodeau 2005; Sherer 1992). In order to achieve the good performance and the strong reliability of automatic gender recognition system, some hybrid algorithms and new gender features should be taken into considered. According to its inherent uniqueness, versatility, and ability to resist deception, physiological measurements can be adopted to study and analyze the gender recognition from some biomedical signals such as blood, heart and brain. Pham et al. (2014) explored the potential of using EEG for user authentication by taking the advantage of rich information including age and gender, and adopting autoregressive features and power spectral density features for analyzing EEG signals in multi-level security systems, which showed a very promising result with a recognition rate of 97.1% for gender authentication. Bilalpur et al. (2017) examined the utility of implicit user behavioral signals including EEG brain signals and eye movements for gender and emotion recognition based on ERP analysis and eye-tracking analysis, obtained a peak AUC of 0.714 for gender recognition. Due to the fact that the analysis and research of these signals is very important for gender recognition, previous studies have shown that the potential effectiveness of biomedical signals in the gender determination was proved by the obvious gender differences existed in physiological measurements. Xue and Farrell (2014) discussed some major gender differences in 12 lead ECG measurements based on automatic algorithms including global measurements and lead-by-lead measurements, obtained a high specificity of 98%. Ku et al. (2012) extracted HRV features in a gender classification system based on ECG signals from 12 samples and obtained an accuracy of 92% by LS-SVM classifier. In another work, Borghetti et al. (2006) studied a face gender recognition tasks based on EEG analysis. Phung et al. (2015) used conditional entropy approach as a feature extraction method for multi-channel EEG-based person identification, compared with the baseline Autoregressive modelling method and achieved a higher identification rate. Nguyen et al. (2013) developed an automatic age and gender recognition system based on EEG signal from 40 samples, achieved a near accuracy of 97% by using SVM classifier. Since EEG is a direct response to the state of the brain, it helps to establish an automatic gender recognition system based on individual signal characteristics (Arya et al. 2013).However, few studies have employed the hybrid model method based on EEG signals to study gender recognition, which may be a promising application of EEG-based systems for assessing and analyzing user gender in biometric authentication systems.

In recent years, various machine learning techniques have been expanded and applied widely in several different fields, which more or less show the strengths and weaknesses of their individual application (Mu et al. 2017; Hu et al. 2015; Kotsiantis 2007; Hu 2017; Min et al. 2017). The random forest (RF) classifier is likely to be a better fit for problems with a small number of features and plenty of training examples. In such a case, variance is a smaller concern and one would likely be better off opting for RF with low bias. The RF assumes the splits are axis-parallel and will become more complex with the increase in the number of features and multiple decision boundaries are possible. On the other hand, logistic regression (LR) assumes there is only one decision boundary that is smooth and non-linear. The RF constructs decision boundaries as follows: (1) selecting the best attribute/feature to divide a set at each branch; and (2) deciding whether each branch is justified adequately. The LR constructs decision boundaries as follows: (1) stepwise selections of the variables and the corresponding coefficients computed; and (2) The maximum-likelihood ratio is used to determine the statistical significance of the variables which will be part of the LR equation. A complex RF may over-fit the data and trees will become unstable (Anděl et al. 2015). With very high dimensional (and possibly sparse) features, LR regularization is critical to avoid over-fitting (Huttunen and Tohka 2015).

Using RF over LR is suitable when their performance is equal and the additional accuracy of RF does not weigh on the increased complexity of the implementation of the model. He et al. (2014) applied the Decision Tree (DT) + LR and Gradient Boost Decision Tree (GBDT) + LR model with Facebook data and found that it worked well. In this study, we explore the application of RF + LR in gender recognition, and determine if it is possible to further improve the recognition performance, and if there is a certain robustness.

**Fig. 1:**
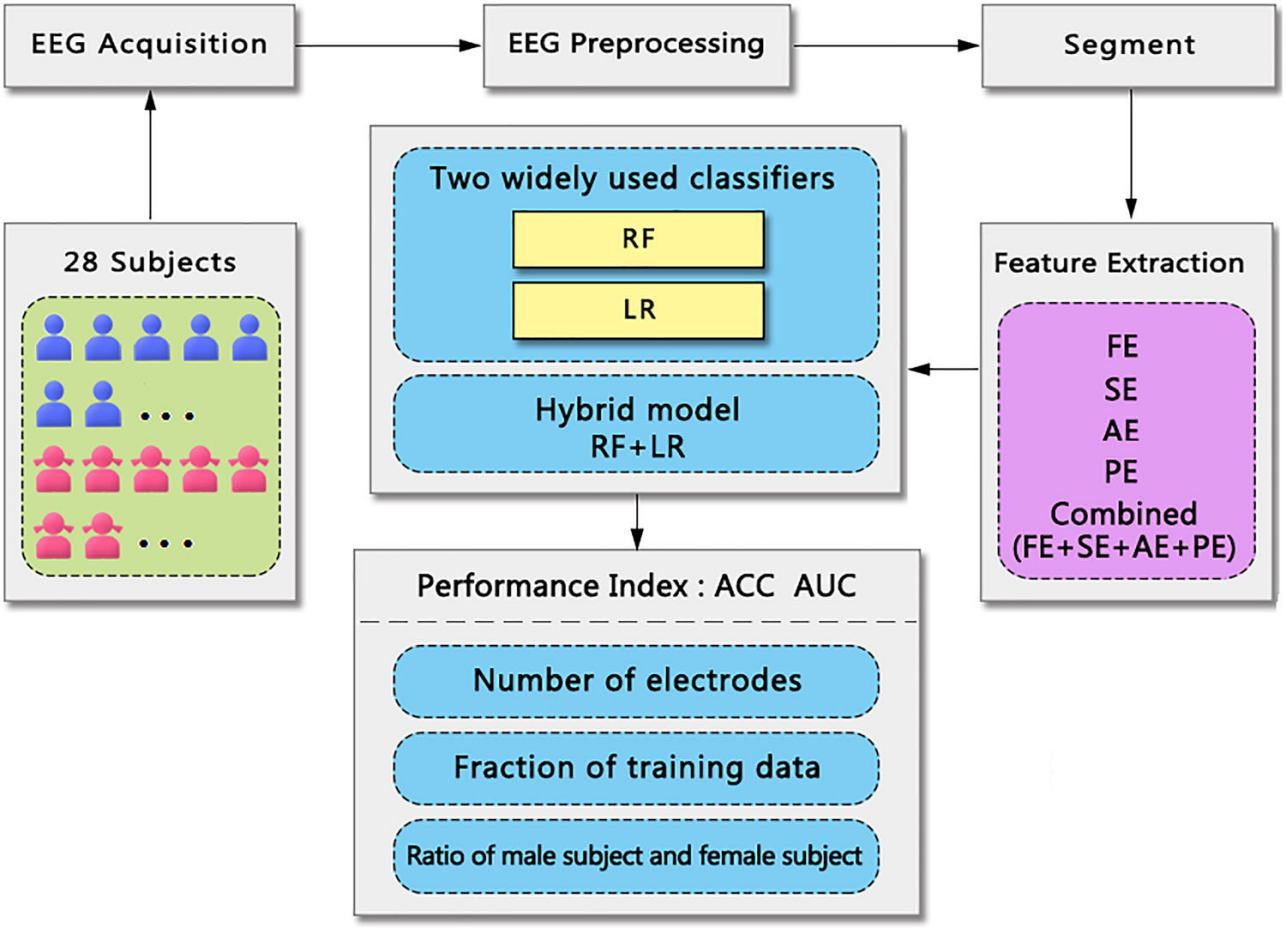
A workflow of the proposed study

In this paper, we present an EEG-based biometric authentication system to effectively access and analyze personal gender by applying a hybrid model combining RF over LR based on four common entropy measures, i.e., FE, SE, AE and PE. According to the influence of the number of electrodes and the fraction of training data, we evaluated and analyzed this hybrid model by using thirty electrodes to improve the authentication results. In addition, this approach was founded on a nested tenfold cross-validation loop that embedded an inner tenfold cross validation for determining parameters that would yield the best classification performance in detector design. Twenty-eight healthy participants performed continuous rest-EEG experiment. The nested 10-fold cross-validation method obtained an accuracy of 0.9982 and an AUC of 0.9926 in average. The EEG-based biometric authentication system is of potential benefit for medical diagnosis and information security in relevant areas and may have a complementary role in existing methods. Figure 1 shows the operation process of an EEG-based gender recognition systems, which primarily includes EEG acquisition, EEG preprocessing, segment, feature extraction, and classification and results analysis.

## Materials and methods

### Subjects

Twenty-eight healthy participants (13 males and 15 females, 18–30 years old), were enrolled in the gender recognition experiment. They were in good health and had not substance abuse. Meanwhile, each participant was not allowed to have the stimulant drink such as tea or alcohol on the day before the experiment. In addition, all subjects understood the experimental procedures and purposes. It should be specially explained that this gender recognition experiment based on multi EEG channels could not cause any damage to the human body, approved by the academic ethics committee of the Jiangxi University of Technology and provided the hand-written informed consent by each subject.

### Data recording and preprocessing

**Fig. 2:**
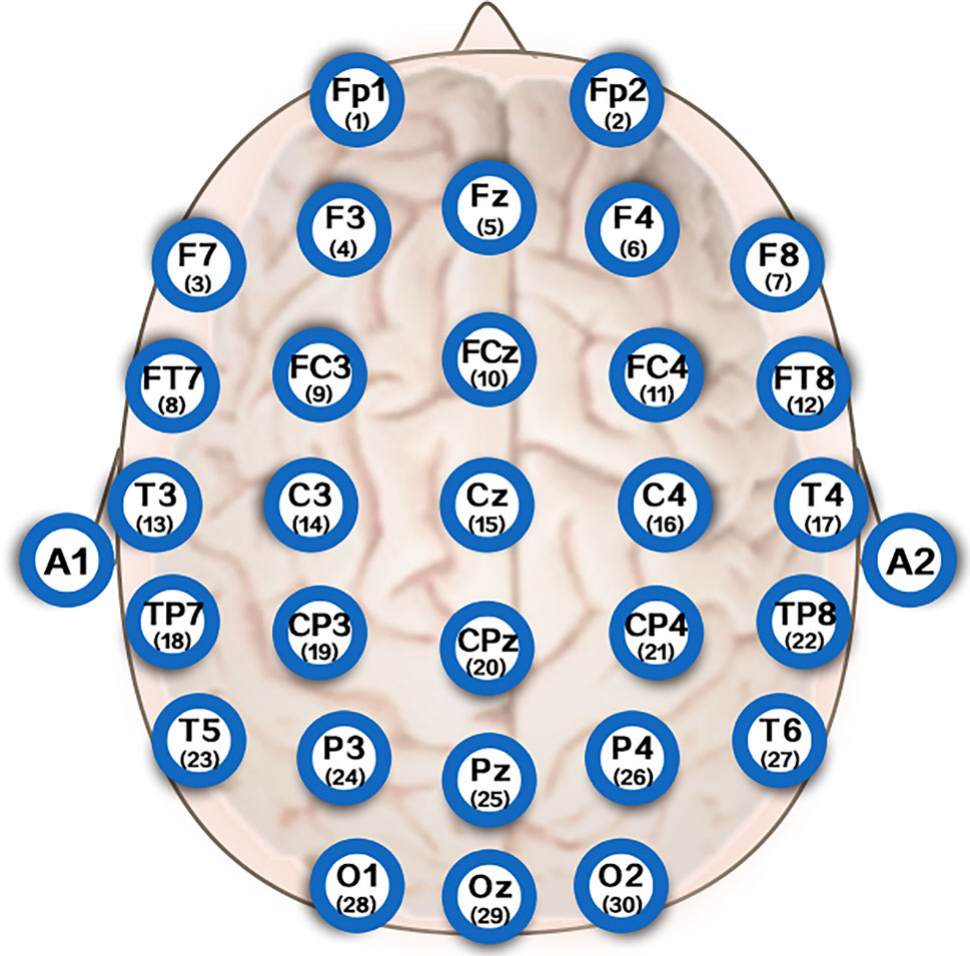
Electrodes position according to the international 10–20 system standard

The subjects sat in a quiet room, free from sound and electromagnetic interference. They kept their eyes open and did nothing, staying awake for 20 min. The EEG signals of the first few minutes in the recording process were discarded due to the fact that the subjects needed some time to calm down completely. Thus the EEG data was recorded in the last 5 min and it was labeled as the dataset in this study. Meanwhile, EEG data from all electrodes were referenced to two electrically linked mastoids at A1 and A2, digitized at 1000 Hz from the 32-channel electrode cap (including 30 effective channels and two reference channels), according to the international 10–20 system, as shown in Fig. 2.

After the EEG signals were collected, the main steps of data preprocessing were implemented by a comprehensive EEG/ERP acquisition and analysis software (Neuroscan scan 4.5, Compumedics in Australia). It should be pointed out that the original EEG was firstly filtered with a 50 Hz notch filter, and then a 0.15–45 Hz band pass filter was used. Next, the EEG signals of the recorded 5 min were segmented into one-second epoch, resulting in 300 epochs for each electrode. Then, a total of 8400 epochs of datasets from 28 subjects were fully obtained at all electrodes. Finally, a total of 8400 (300 × 28) units were randomly constructed for training and testing datasets, in which 4500 units were available for females and 3900 for males.

### Feature extraction

Though EEG is assumed to be a non-stationary time series, most feature extraction methods are only applicable to stationary signals. Some researchers have also used power spectrum density (PSD) and autoregressive (AR) models, but these methods are difficult to use with non-stationary EEG signals. To solve this problem, the EEG data is split into several short windows, and the statistics are assumed to be approximately stationary, just like many articles used this same method (Min et al. 2017). The following feature extraction method is applied to each of the one-window signals. The EEG signals are segmented without overlapping, and finally all the electrodes in each 1-s window are extracted from the feature set.

As the nonlinear parametric, entropy factors have been widely used to assess the uncertainty of a system. Due to the fact that entropy evaluators can quantify the complexity of a time series degree, which can be used to describe non-linear, unstable dynamic EEG signal, have been broadly applied in recent years (Acharya et al. 2012; Mu et al. 2016; Phung et al. 2014). A variety of different methods of collection have been proposed in the last few decades, including fuzzy entropy (FE), sample entropy (SE), approximate entropy (AE), permutation entropy (PE), information entropy, Renyi entropy, and others. Specifically, in the field of EEG signal processing, the four most widely used entropy estimators are FE (Chen et al. 2009), SE (Richman and Moorman 2000), AE (Pincus 1991) and PE (Reyes-Sanchez et al. 2016). It is worth pointing out that the two parameters *m* and *r* in FE, SE and AE should be considered, which described respectively the dimensions of phase space and similarity tolerance in entropy measures. In this article, we adopted *m* = 2 and *r* = 0.25*SD (*SD* denotes the standard deviation) according to the literatures (Yentes et al. 2013). For optimizing the detection quality, the features were normalized for each subject by scaling between − 1 and 1 based on the min–max normalization after building a feature vector via the concatenation process.

The ability to distinguish between men and women depends largely on the quality of the input vectors in the classifier. To capture gender-related EEG features, four feature sets were measured, including FE, SE, AE and PE, and using a combination feature set (FE + SE + AE + PE). In this section, the calculation method of the entropy set is described in detail (Mu et al. 2016, 2017; Hu 2017; Hu and Wang 2017).

### Classification

#### Logistic regression (LR)

As a generalized linear model, LR is widely used in various fields including machine learning and most medical fields, which describing the probability of a binary response based on one or more predictor variables by using a logistic function (Prasad et al. 2014). Two main parameters require tuning: penalty and C. Parameter penalty is used to specify the norm used in the penalization. C represents the inverse of the regularization strength, and smaller C values specify stronger regularization. In this work, penalty is l1 regularization and C is 1.0, unless otherwise stated.

#### Random forest (RF)

RF is defined as a group of unpruned classification or regression trees, trained on bootstrap samples of the training data using randomly-selected variables or features in the process of tree generation (Surhone et al. 2010). RF fits a number of decision tree classifiers on various sub-datasets and averages the predicted accuracy. Two parameters require tuning: number of trees (nt) and the max depth of the tree (md). In this work, the md is 5 and nt is 200, unless otherwise stated.

#### Hybrid model

**Fig. 3:**
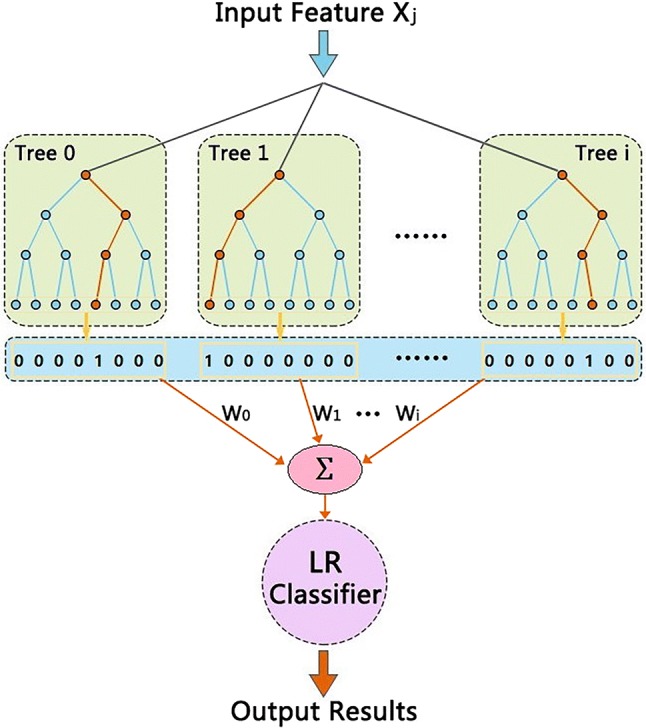
Proposed hybrid model structure. Input entropy values are transformed to sparse vectors that can been created by subtrees of RF. The output of each subtree is treated as a categorical input feature to LR classifier. Here, Xj represents the j-th entropy feature vector, Treei (i = 0, 1,…) is the i-th subtree constructed by RF model and Wi is the weight value of the i-th subtree in RF model

After building entropy feature vectors, the concatenation of the RF and LR classifiers should be established, shown in Fig. 3. First, we fit a random forest on the training set via inputting the feature vectors. Then, sparse vector from each leaf of subtree in RF model is assigned a fixed arbitrary feature index in a new feature space. The RF classifier can transform the feature vectors into a higher dimensional and sparse feature space. Thus these leaf indices are encoded in a one-shot fashion according to the input entropy features. Each sample is encoded by setting the feature values for these leaves to 1 and the other feature values to 0, while the weight Wi can been optimal via cross validation. Finally, we train a LR model on the combination of these sparse features and original features. A hybrid method is being built in this paper, which makes use of ensemble learning from RF and LR classification method.

### Parameter setting

A multi-classifier optimizing strategy is an important part in machine learning, and there are many methods to reach and evaluate the best optimized performance. The classifier, in general, has two types of parameters: a class of parameters from the data can be estimated by learning processes, and other parameters cannot be estimated from the data, which are called hyperparameters. For example, penalization and regularization strength for LR, the number of trees, the number of features, and maximum depth of the tree for RF. In this study, a grid hyperparameter search was used to achieve better results vid an inner 10-fold cross-validation approach. A grid hyperparameter search usually consists of several parts: a classifier, parameter space, search mechanism, cross validation, and performance function. In this article, the performance function is used directly with the accuracy rate. By testing in advance to reduce the parameter space range, and then small steps and a global search were used, generating the test set and training set reasonably. According to different analysis objectives, special optimization was performed for different subjects and/or different feature sets, and so on.

### Performance metrics

**Fig. 4:**
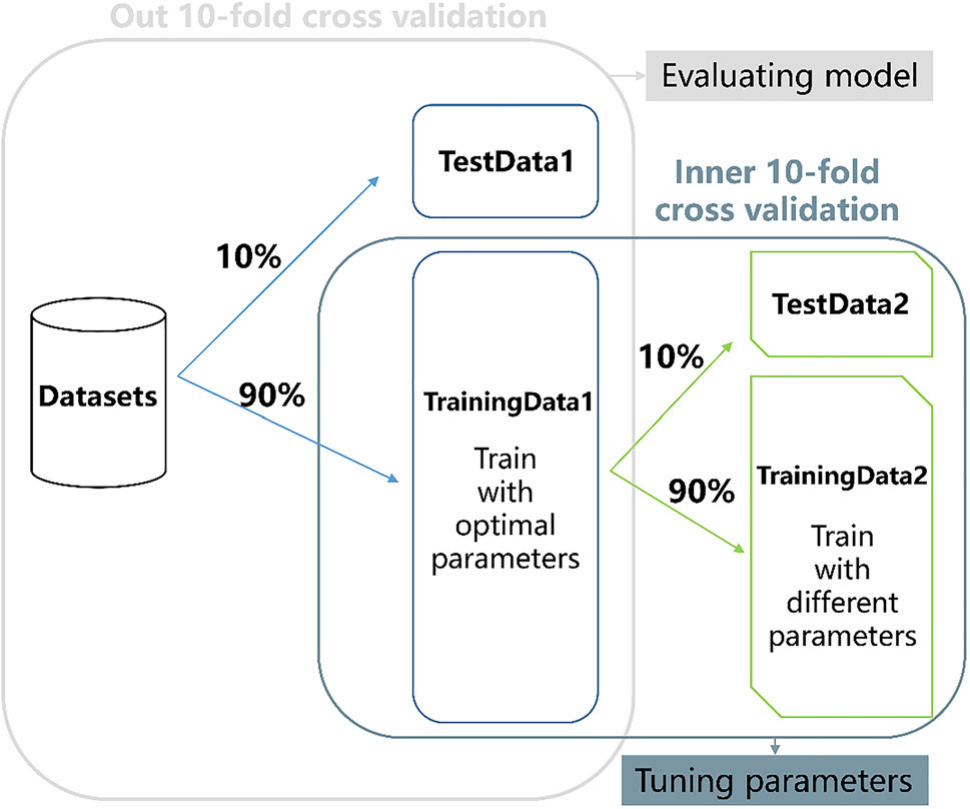
A nested 10-fold cross-validation loop

In order to assess the potential application performance of a sex detector, it is important to correctly compare the quality of the detector and weight the phenomenon of overfitting. Therefore, a nested 10-fold cross validation loop is used to evaluate the performance of the gender identification system, where 10% of dataset defined as test dataset while the remaining subjects used to train with model evaluation in outer loop and an inner loop for tuning parameters, see Fig. 4. In the next iteration, another 10% of dataset feature vectors are considered as test sets, and the rest are training sets until all the feature vectors are involved in the test phase. The final result is achieved by averaging the results produced at the corresponding corners. In addition, performance indicators include the average accuracy and the curve area under the receiver operating characteristic curve (ROC curve) for performance evaluation. The average accuracy of this paper refers to the average recognition rate of all subjects of a feature set and/or classifier. The AUC illustrates the performance of the two-classifier system because its discriminant threshold is variable.

### Calculation tool

We used the scikit-learn toolbox (Pedregosa et al. 2011) to train and test. The regularization parameter was optimized using an inner 10-fold cross-validation procedure. For example, RF models were trained with the sklearn.ensemble.RandomForestClassifier module of the Python library scikit-learn using the following parameters: (1) number of trees in the forest; (2) criterion to assess the quality of a split; (3) minimum number of data points to split a node; and (4) minimum number of data points in a leaf to keep a given node split. LR models were trained using the sklearn.linear_model.LogisticRegression module of the Python library scikit-learn using the following parameters: (1) tolerance for stopping criteria; and (2) inverse of regularization strength.

## Results

The main idea behind this paper was to find a more efficient method for gender recognition, and to explore the hybrid model combining RF and LR.

### Comparison of entropy between male and female

**Fig. 5:**
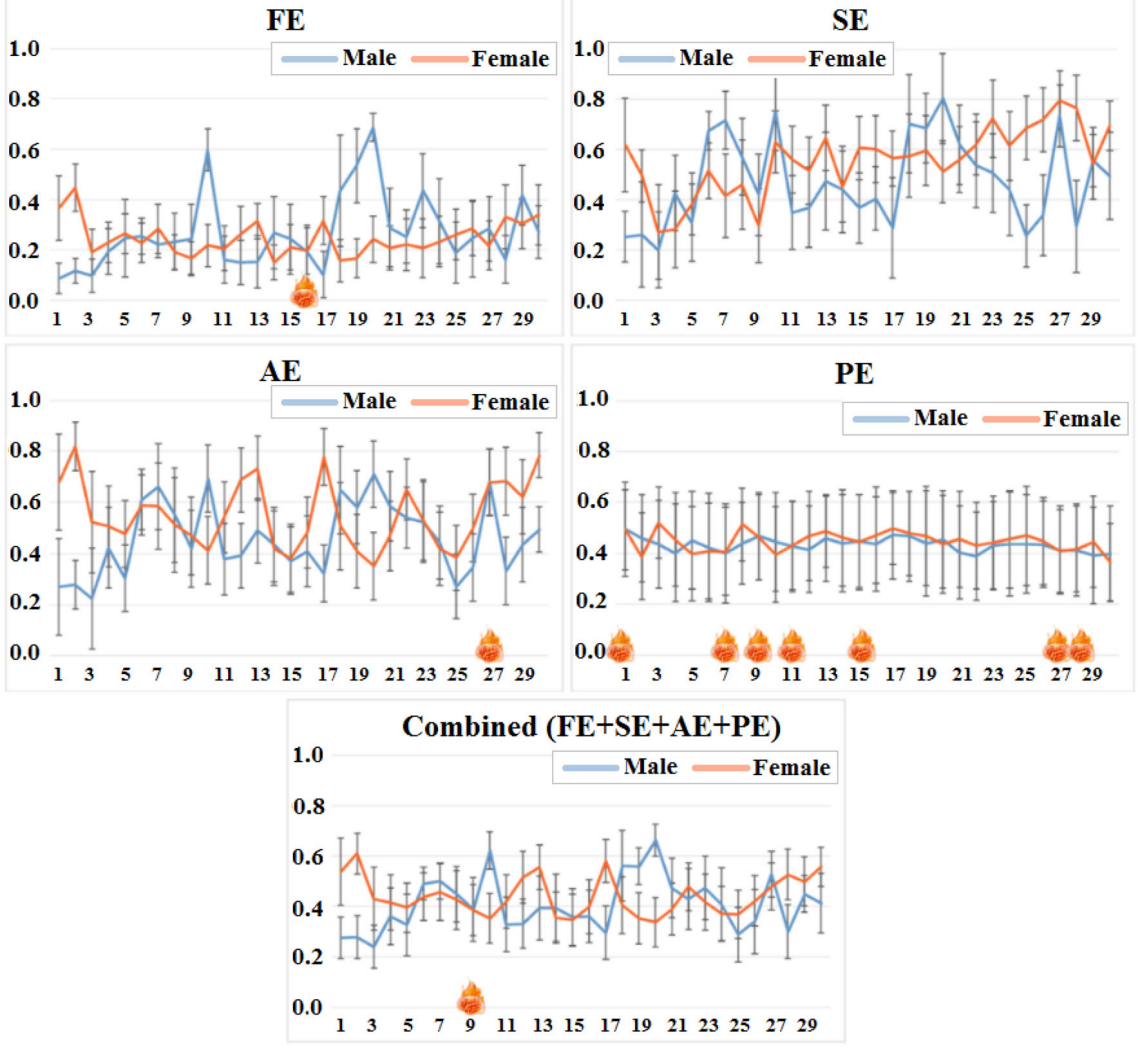
Entropy feature comparison for male and female according to EEG channels (shown in Fig. 2). The vertical coordinate represents the magnitude of entropy, while the horizontal coordinate is the index of EEG channels.  represents that the difference is not significant (*p* > 0.01)

In this study, for evaluating the performance influence on different entropies for personal gender, we calculated the values of different entropies between PE, AE, SE and FE as features according to EEG channels. The paired-samples *t* test was employed to evaluate the quantified results between male and female. Thus a comparison of different entropy measures between male and female was shown in Fig. 5. As you can see from this figure, there are significant differences for different entropy values from EEG signals between male and female with the electrode subscript changed. For example, for the forehead electrode of great importance (No.1, Fp1), the values of FE from female subjects is significantly higher than that from male subjects (0.37 ± 0.13 vs. 0.09 ± 0.06, *p* < 0.001), SE is 0.62 ± 0.19 vs. 0.25 ± 0.10 and *p* < 0.001, AE is 0.68 ± 0.19 vs. 0.27 ± 0.11 and *p* < 0.001, and the combined entropy (FE + SE + AE + PE) is 0.54 ± 0.13 vs. 0.28 ± 0.08 and *p* < 0.001. However, the values of PE don’t present the difference between female and male subjects. Furthermore, other electrodes also show this gender difference from these four common entropies.

A significant difference in the entropy values was determined based on personal gender between FE, SE, AE and the combined entropy in Fig. 5, which reveals that the entropy meatures can exhibit a good performance for recognizing personal gender. In addition, observation of electrode channels can be found that the top three electrodes from relatively independent area with the largest entropy difference for FE between female and male subjects were No.20 of CPz, No.10 of FCz and No.1 of Fp1, No.28 of O1, No.25 of Pz and No.26 of P4 for SE, No.2 of Fp2, No.17 of T4 and No.1 of Fp1 for AE, and No.2 of Fp2, No.20 of CPz and No.17 of T4 for the combined entropy. Therefore, the forehead electrode Fp1 and Fp2 may have potential applications when we use only one (or two) electrode(s) to detect personal gender in the field of practical applications due to the importance distinction based on different entropies from EEG signals.

### Comparison of classifiers

**Fig. 6:**
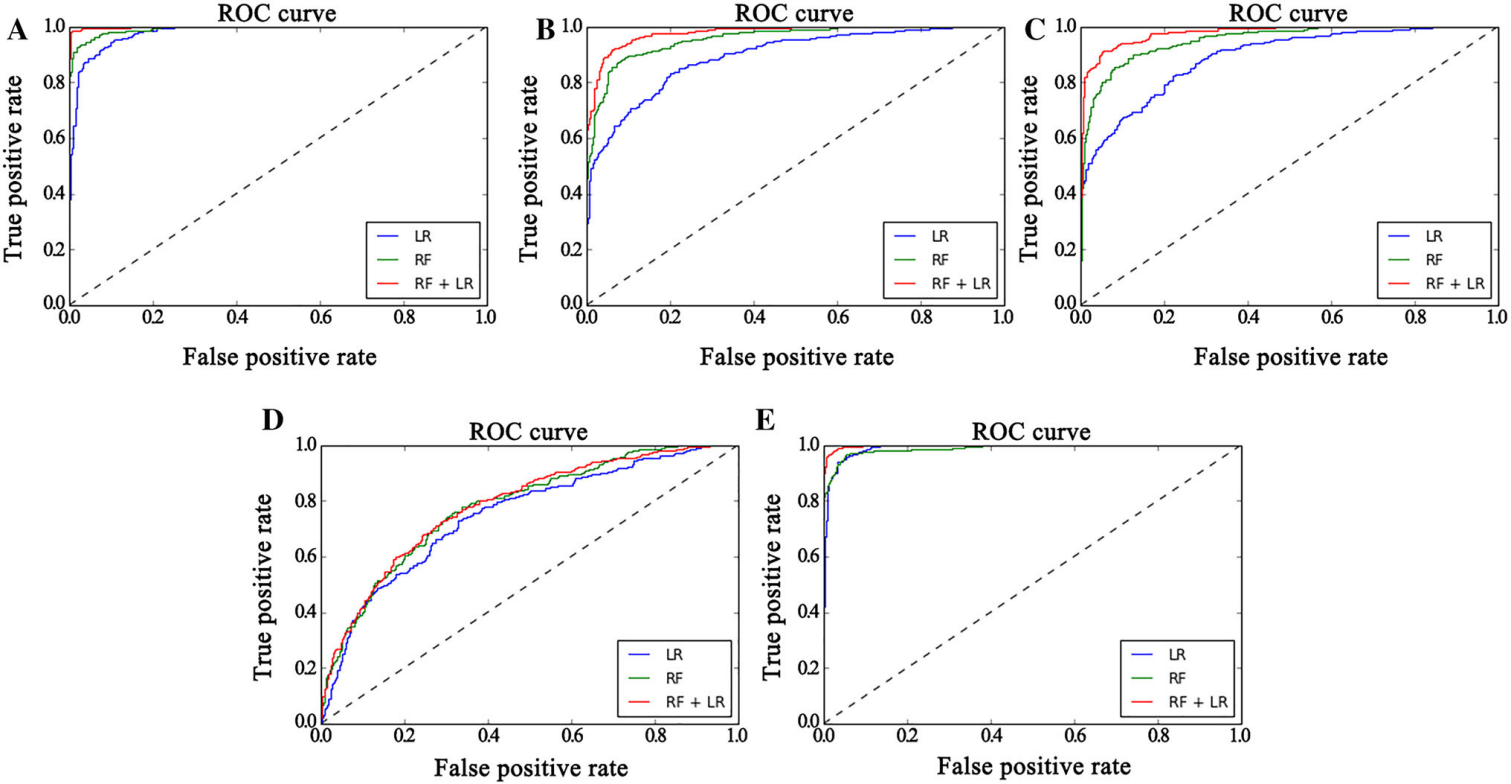
ROC curves for different feature sets and different classifiers. **A**, **B**, **C**, **D** and **E** respectively represent FE, SE, AE, PE and combined feature sets

To show the performance of the hybrid model, this article compares it to the recognition performance of LR and RF. The results of ten independent rounds are used to draw mean ROC curves. Different feature sets or classifiers were compared by analyzing their ROC curves and areas under the ROC curves (AUC). In Fig. 6A–E, their performance in the ROC curves produced was compared by different classifiers on the FE feature set, SE feature set, AE feature set, PE feature set, and the combined entropy feature set, respectively. This shows that the FE feature set and combined feature set outperform other feature sets significantly (paired *t* test, *p* < 0.01). For example, the best AUC of the FE feature set and the combined feature set are 0.9982 and 0.9983, respectively, while the best AUC of the SE, AE, and PE feature sets are 0.9421, 0.9483, and 0.7118, respectively. Consequently, adding more features results in no changes for gender detection. Therefore, the FE feature set is selected for the next experiments.

Taking the FE of the 30 electrodes as the feature sets, with AUC as the index, it can be seen that the performance of RF + LR (0.9982) > RF (0.9926) > LR (0.9801) from Fig. 6A. The other three types of entropy as feature sets, shown in Fig. 6B–D, show similar results, but PE is not significant. Can multiple features improve performance? As shown in Fig. 6E, the four entropy values are simply combined into the combined feature set as input feature sets, and it can be seen that the performance of RF + LR (0.9983) > RF (0.9891) > LR (0.9911). The ACC and AUC of the five entropy feature sets are shown in Table 1, which LR-ACC, RF-ACC and RF-LR-ACC represent the average accuracy of using classifier by LR, RF and RF + LR respectively while LR-AUC, RF-AUC and RF-LR-AUC represent the value of AUC by the above similar classifiers. It can be obviously observed from the table that the classifier by using hybrid model RF + LR can obtain the better classification accuracies based on different entropy feature sets and hit a highest average accuracy of 99.83% by using combined entropy features based on RF + LR classifier. Similarly, it achieved a better performance of the AUC by using a hybrid model RF + LR classifier based on different entropy feature sets. But the highest value of AUC with 0.9926 was reached by using FE feature sets based on RF + LR classifier, which implying that fuzzy entropy could have a significant influence on the gender recognition and maybe superior to or close to the combined entropy features. Furthermore, it could be effective and convenient for just using fuzzy entropy to recognize the gender of persons in practical application.

**Table 1:** Average accuracy and AUC for various classifiers based on different feature sets

	FE	SE	AE	PE	Combined
LR-ACC	0.9801	0.8952	0.8910	0.7508	0.9911
RF-ACC	0.9926	0.9578	0.9539	0.7811	0.9891
RF-LR-ACC	0.9982	0.9785	0.9792	0.7850	0.9983
LR-AUC	0.9187	0.7980	0.7845	0.6847	0.9507
RF-AUC	0.9384	0.8929	0.8818	0.7007	0.9286
RF-LR-AUC	0.9926	0.9421	0.9483	0.7118	0.9717

LR-ACC, RF-ACC and RF-LR-ACC represent average accuracy for LR classifier, RF classifier and RF + LR classifier, respectively. Similarly, LR-AUC, RF-AUC and RF-LR-AUC represent average AUC for LR classifier, RF classifier and RF + LR classifier, respectively

### Effect of parameters

The performance of the classifier model is affected by the parameters, and RF and LR have different parameters. Does the change of parameters of a single classifier affect the performance of the hybrid model? This study explored this.

The main parameters to be adjusted in RF are parameter max depth (*md*) and number of trees (*nt*). The best performance of the RF model can be yielded through carefully choosing the optimal combination of these parameters. The parameter *md* controls the maximum depth of the tree. As shown in Table 2, accuracy of RF reaches the maximum of 0.9409 when *nt* is 50, while the AUC reaches the maximum of 0.9931 when *nt* is equal to 20, accuracy of hybrid model reaches the maximum value of 0.9915 when *nt* is 2000, and AUC of hybrid model reaches the maximum value of 0.9986 when *nt* is equal to 1000. As shown in Table 3, the larger the *md*, the greater the ACC and AUC. When *md* equals 20, both the ACC and AUC of RF reach the maximum, while AUC of hybrid model also reaches the maximum, and ACC of hybrid model reaches the maximum when *md* equals 7. As mentioned above, the hybrid model does not require larger *md*, and the larger depth does not increase the hybrid model’s ACC and AUC, although the larger *md* will increase the RF performance.

**Table 2:** Influence of the parameter *nt* of RF classifiers on the average accuracy for three classifiers with FE feature set

*nt*	1	10	20	50	100	200	500	1000	2000
RF-ACC	0.8670	0.9236	0.9384	**0.9409**	0.9360	0.9384	0.9323	0.9347	0.9335
RF-LR-ACC	0.6256	0.9052	0.9470	0.9754	0.9840	0.9926	0.9963	0.9963	**0.9975**
RF-AUC	0.9304	0.9871	**0.9931**	0.9930	0.9920	0.9926	0.9925	0.9926	0.9925
RF-LR-AUC	0.9322	0.9930	0.9971	0.9969	0.9975	0.9982	0.9983	**0.9986**	0.9985

Bold indicates the highest recognition rate in this row

RF-AUC and RF-LR-AUC represent average AUC for RF classifier and RF + LR classifier, respectively. RF-ACC and RF-LR-ACC represent average accuracy for RF classifier and RF + LR classifier, respectively

**Table 3:** Influence of the parameter *md* of RF classifiers on the average accuracy for three classifiers with FE feature set

*md*	1	2	3	4	5	6	7	8	9	10	20
RF-AUC	0.9194	0.9410	0.9644	0.9824	0.9926	0.9969	0.9990	0.9995	0.9998	0.9998	**0.9999**
RF-LR-AUC	0.9358	0.9879	0.9946	0.9970	0.9982	0.9989	0.9988	0.9993	**0.9995**	0.9994	**0.9995**
RF-ACC	0.8534	0.8941	0.9064	0.9175	0.9384	0.9532	0.9680	0.9766	0.9828	0.9865	**0.9938**
RF-LR-ACC	0.9483	0.9741	0.9852	0.9865	0.9926	0.9889	**0.9963**	0.9926	0.9914	0.9951	0.9938

Bold indicates the highest recognition rate in this row

RF-AUC and RF-LR-AUC represent average AUC for RF classifier and RF + LR classifier, respectively. RF-ACC and RF-LR-ACC represent average accuracy for RF classifier and RF + LR classifier, respectively

As shown in Table 4, whether the value of penalty is l1 or l2, and the effect on the LR and the hybrid models are not significant, but the value of C has a greater impact on the performance of recognition.

**Table 4:** Influence of the parameters (penalty and C) of LR classifiers on the average accuracy for three classifiers with FE feature set

Penalty	C	LR-AUC	RF-LR-AUC	LR-ACC	RF-LR-ACC
l2	10	0.985	0.996	0.936	0.973
l2	1	0.980	**0.997**	0.919	0.975
l2	0.1	0.960	**0.997**	0.884	0.968
l2	0.01	0.929	0.994	0.867	0.948
l2	0.001	0.917	0.983	0.860	0.903
l1	10	**0.986**	0.993	**0.942**	0.972
l1	1	0.985	0.994	0.936	**0.977**
l1	0.1	0.971	0.984	0.909	0.957
l1	0.01	0.910	0.818	0.839	0.887
l1	0.001	0.500	0.500	0.528	0.528

Bold indicates the highest recognition rate in this column

LR-AUC represents average AUC for LR classifier and RF-LR-AUC represents average AUC for RF + LR classifier. LR-ACC represents average accuracy for LR classifier and RF-LR-ACC represents average accuracy for RF + LR classifier

### Comparison of number of electrodes

Is it possible to achieve satisfactory performance with fewer electrodes and enough features? To explore the effect of the number of electrodes for the detection system, we evaluated the system performance with respect to the number of electrodes. For each number m (from 1 to 30), a random combination (m out of 30 channels) was repeated 10 times to calculate the classification accuracy, and the accuracy was averaged.

**Fig. 7:**
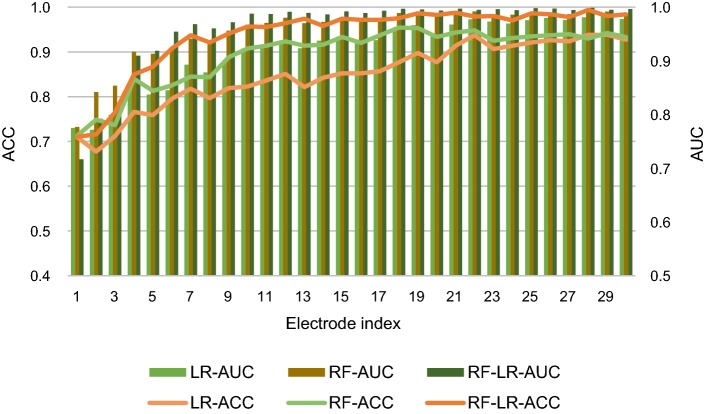
Average accuracy with cumulative electrodes based on FE feature set. LR-ACC, RF-ACC and RF-LR-ACC represent average accuracy for LR classifier, RF classifier and RF + LR classifier, respectively. Similarly, LR-AUC, RF-AUC and RF-LR-AUC represent average AUC for LR classifier, RF classifier and RF + LR classifier, respectively. Electrode index is shown in Fig. 2

As shown in Fig. 7, the variation trend of the average accuracy of different classifies is similar. For example, for the classifier LR, the average accuracy basically increases (*p* < 0.01) with the number of electrodes increasing. In other words, more electrodes do improve the performance significantly.

The greater the number of electrodes, the more feature sets, and the accuracy may be more ideal. However, a greater number of electrodes will increase the computational complexity and the discomfort of subjects. If possible, under the premise of maintaining a certain performance, as few electrodes as possible should be used.

### Effect of fraction of the training data

**Fig. 8:**
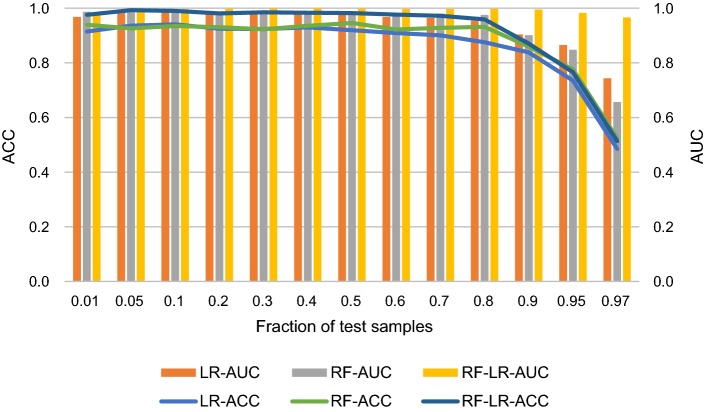
Performance evaluation with respect to the ratio of test samples for all samples based on FE feature set. LR-ACC, RF-ACC and RF-LR-ACC represent average accuracy for LR classifier, RF classifier and RF + LR classifier, respectively. Similarly, LR-AUC, RF-AUC and RF-LR-AUC represent average AUC for LR classifier, RF classifier and RF + LR classifier, respectively

The ratio of test samples to all samples is important for the performance of the classifier. To determine the robustness of the classifier against the size of the test sample, the ratio of test samples to all samples is set varying from 0.01 to 0.97. The ACC and AUC of different classifiers based on the FE feature set against different ratios are shown in Fig. 8. When the test sample is small, there will be more training samples, learning will be more complete, such as changes in the range of 0.01–0.8, and ACC and AUC are not obvious; on the other hand, when testing more samples, there will be fewer training samples, the lack of learning can affect performance, such as in the range of 0.9–0.97, and ACC and AUC decline significantly. The performance of the three classifiers is similar.

### Effect of ratio of male subjects and female subjects

The number of subjects is an important parameter in the gender detection system. More subjects can provide more information that may improve or reduce detection performance. Generally speaking, when average performance is poor, any subject with higher accuracy can improve the overall performance, and vice versa. Sometimes, the classifier model that is suitable for small samples may lose performance when large samples are used. However, when more subjects are involved, the system costs, including hardware and computation time, will also increase. Therefore, a tradeoff between the system performance and system cost should be based on the specificity of the application. In addition, the proportion of male and female samples may also affect the performance, when the male and female samples less samples, or male samples and less female samples, or both samples almost, whether the performance of gender recognition have similar effects, this study designed 27 specific cases, both the total number of samples, but also considering the gender proportion of the sample, the 27 fraction are {13:1, 13:2, 13:3, 13:4, 13:5, 13:6, 13:7, 13:8, 13:9, 13:10, 13:11, 13:12, 13:13, 13:14, 13:15, 12:15, 11:15, 10:15, 9:15, 8:15, 7:15, 6:15, 5:15, 4:15, 3:15, 2:15, 1:15}. For each fraction, a random combination was repeated 10 times for calculating classification accuracy. Three classifiers approaches were calculated for comparison.

**Fig. 9:**
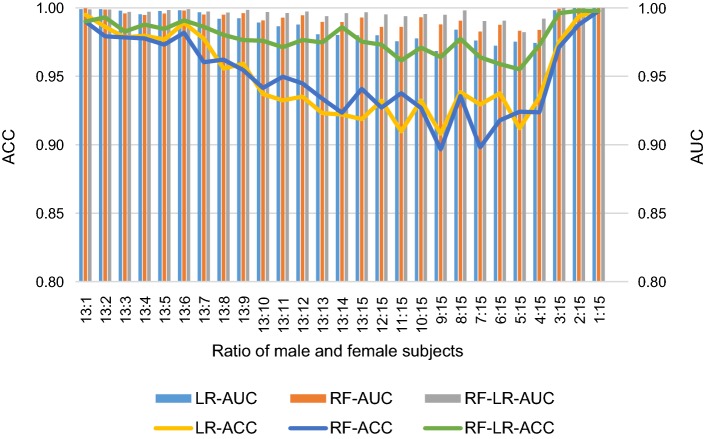
Performance evaluation with respect to the ratio of male subjects and female subjects based on FE feature set. LR-ACC, RF-ACC and RF-LR-ACC represent average accuracy for LR classifier, RF classifier and RF + LR classifier, respectively. Similarly, LR-AUC, RF-AUC and RF-LR-AUC represent average AUC for LR classifier, RF classifier and RF + LR classifier, respectively

In Fig. 9, it can be seen that with the increase in the number of samples, the ACC and AUC of RF and LR decreased quickly, while the hybrid model changes little, such as the 13:1 three LR RF, RF-LR classifier, ACC (AUC) were 0.9951 (0.9991), 0.9901 (0.9997), 0.9901 (0.9989), and the 13:15 is 0.9187 (0.9801), 0.9409 (0.9930), 0.9754 (0.9969); and 1:15, three kinds of classifiers of ACC (AUC) were 0.9978 (1.0000), 0.9978 (1.0000), 0.9978 (1.0000). The effect of the male to female ratio appears to be a U-type change. In the case of more males and females, the ACC of the three classifiers did not differ significantly, and the ACC of the three classifiers decreased significantly when the ratio of male and female approached. The hybrid model is less affected, and effect of RF and LR are about the same.

## Discussion and conclusion

The strategy of using a hybrid model via combining random forest and logistic regression classifiers based on four common entropy factors can remarkably improve the recognition quality of a predictor for person’s gender, as indicated by the nested cross-validation tests in which a wider pool of participants was examined. The hybrid model was initially started by training several trees for each instance and then obtained the prediction results with the leaf index for each tree. Finally, the LR classifier was continued to train based on the binary data of leaf index in hybrid model.

Put in the context of RF + LR, what are the assumptions made? RF assumes that decision boundaries are parallel to the axes. Thus, RF chops up the feature space into higher dimensions, which can create a problem with over-fitting. Despite the probabilistic framework of LR, LR assumes that there is one smooth linear decision boundary. It finds that linear decision boundary by making assumptions that the P(Y|X) of some form, like the inverse logit function, is applied to a weighted sum of our features. Then it finds the weights by a maximum likelihood approach. Thus, if you have data where the decision boundary is not parallel to the axes, then LR picks it out rather well, whereas RF will have problems. LR will work better if there is a single decision boundary, not necessarily parallel to the axis. RF can be applied to situations where there is not just one underlying decision boundary, but many, and will work best if the class labels roughly lie in hyper-rectangular regions. LR is intrinsically simple: it has low variance and, thus, is less prone to over-fitting. RF can be scaled up to be very complex, are more liable to over-fit.

In this paper, an objective approach based on entropy feature sets and various classifiers was proposed to recognize gender in EEG-based systems and the results demonstrated its promise as a method to identify gender by achieving higher success rates. In addition, the related classification performance adopted in some previous studies are listed in Table 5. Compared with other existing EEG-based methods, the proposed method for gender authentication achieved an improved performance and a more robust detector, for studying personal gender conveniently and effectively. It also shows a significant potential applicability of the proposed approach and is capable of identifying personal gender in an EEG-based biological recognition system.

**Table 5:** A performance comparison with the previous works

Authors	Feature set	Classifier	Stimulus type	Sample size	Accuracy (%)
Phung et al. (2014)	AR and PSD	SS2LM-SVDD	Rest	40	97.1
Ku et al. (2012)	HRV	LS-SVM	Rest	12	92.0
Nguyen et al. (2013)	Conditional entropy	SVM	Rest	40	97.0
Hu et al. (2015)	AR	BP	Visual stimuli	15	92.9
Phung et al. (2014)	Shannon entropy	SVM	Rest	40	94.9
Maiorana et al. (2016)	PSD	Nearest-neighbor	EC and EO	30	87.9
This paper	Four entropies	A hybrid model	Rest	28	99.8

With the purpose of providing a more efficient method for recognizing gender, a hybrid model, LR classifier, and RF classifier were compared. It was found that: (1) It is possible to use EEG signals for gender recognition. The highest recognition rate in this work could reach 0.9982 accuracy based on a combination of FE and hybrid models, which could meet the needs of daily applications. (2) The effect of parameters of single classifiers on the hybrid model (RF +LR) is smaller than that of a single classifier (RF or LR). (3) The number of electrodes has great influence on the performance of the classifiers, and the influence of a single classifier (RF or LR) is greater than that of the hybrid model. (4) The number of training samples has an impact on the recognition effect, but fewer training samples can also achieve satisfactory results. (5) For the recognition rate, a different fraction of male subjects and female subjects have different effects.

In conclusion, EEG-based biological gender recognition by using different entropies has a potential application for information safety and clinical research, referred to social emotion, person identification, treatment uptake, clinical efficacy and adverse reactions (Freeman et al. 2017; Shearer et al. 1984). For example, Thul et al. adopted Permutation entropy and Symbolic Transfer entropy to analyze EEG signals for clinical assessments, which implying that the utilized EEG entropy analyses were able to relate to patient groups with different disorders of consciousness (Thul et al. 2015). Jausovec et al. concluded that males and females differ in the local as well as long range coding of information as well as in the excitability dynamics of their cortical network by using the total power, coherence and approximate entropy measures from EEG signals (Jausovec and Jausovec 2010). In addition, some cutting-edge technologies should be benefit for promoting the robustness and stability of the recognition performance for personal gender, especially used of deep learning technique (Faust et al. 2018). In this work (Mamun K 2017), deep learning had been used for MI EEG signal classification. Robin et al. studied deep learning with convolutional neural networks for EEG decoding and visualization, which boosted the deep convnets decoding performance. Other biological clinical researches such as seizure diagnosis also adopted deep learning techniques (Acharya et al. 2017), which can be very useful for us to learn in the following research work.

However, some limitations of this study were: (1) The sample size was small, with only 28 subjects and 504,000 units for 30 electrodes. To extend our research, the number of subjects should be increased to improve the validation of results. (2) Only four entropy feature sets and a combined feature set were compared in this study, some other entropy measures such as Wavelet entropy, Permutation entropy, HOS phase entropy, Bispectrum entropy and Tsalli’s entropy also have been widely used and should be studied further for EEG-based gender recognition in our ongoing researches. (3) Different brain areas may have different gender characteristics so the classifiers should be area-specific or subject-specific. (4) This study used EEG signals in the resting state, so specific tasks, such as movement imagination, event-related potentials (ERPs), and other behaviors that may make gender differences more pronounced may achieve better performance. (5) The effects of various parameters on the hybrid model need to be further investigated, and these will be explored in future research. (6) One problem with the proposed approach using EEG signals is that these signals cannot be easily acquired in an unobtrusive way. In other words, subjects need to wear sensors to acquire data. The invasiveness makes such signals difficult to acquire and are not practical for real-time applications. For this actual application, an offline analysis on EEG datasets was performed and recorded from online experiments in this study. However, owing to the fact that the offline and online classifications have distinct characteristics, a further study in a real-time online experimental environment should be conducted to confirm the present findings. It is suggested that a real-time gender recognition system with wireless EEG device such as smartphone, tablet and cloud server could be widely used in the future. Thus it is necessary to build a platform for mobile gender identifying system meeting the requirements of real-time online modality. A global sensitivity and uncertainty analysis of personal identifying model/system would be useful to capture the robustness of the identification results in the future (Convertino et al. 2014). Further research may solve these problems and lead to a better approach to EEG signal-based gender classification.
